# Multidrug-Resistant Acinetobacter: Detection of ESBL, MBL, *bla*_*NDM-1*_ Genotype, and Biofilm Formation at a Tertiary Care Hospital in Eastern Nepal

**DOI:** 10.1155/2022/8168000

**Published:** 2022-12-10

**Authors:** Manisha Kumari, Narayan Raj Bhattarai, Keshav Rai, Tejendra Kumar Pandit, Basudha Khanal

**Affiliations:** Department of Microbiology, B. P. Koirala Institute of Health Sciences, Dharan, Nepal

## Abstract

**Background:**

The Acinetobacter species is an important hospital-acquired pathogen. The rapid development of resistance to multiple drugs and the ability to form biofilm make these bacteria more adaptable to survive in healthcare facilities, thus posing a challenge to their effective management.

**Objective:**

This study aimed to characterize clinical isolates of Acinetobacter spp and to study their antimicrobial susceptibility patterns and ability to form biofilm. Resistant Acinetobacter was further analyzed for the detection of extended-spectrum *β*-lactamases (ESBLs), metallo *β*-lactamases (MBLs), carbapenemase production, and presence of blaNDM-1 gene.

**Materials and Methods:**

A total of 324 Acinetobacter species were isolated from various clinical specimens which were submitted to the Department of Microbiology, B.P. Koirala Institute of Health Sciences, Dharan, Nepal, and were studied for antibiotic susceptibility testing, detection of ESBL and MBL production, and formerly biofilm formation was performed by standard microbiological methods. PCR was carried out to determine the presence of the blaNDM-1 gene.

**Results:**

The predominant Acinetobacter species isolated was A calcoaceticus-baumannii Complex (Acb complex) 167 (51.5%). Among those, all A. species 128 (40%) were multidrug resistant (MDR). In which 13 (4.0%) were ESBL producers, 70 (61.9%) were MBL, and 12 (10.6%) were carbapenemases producers. The blaNDM1 gene was present in 33 isolates. Thirty-seven percent (121/324) of isolates formed biofilm. The majority of A. species were resistant to cefotaxime 73.8% (239) and cefepime 74.4% (241). A significant proportion of biofilm producers were MDR (*p* < 0.001).

**Conclusion:**

Drug-resistant Acinetobacter formed a substantial proportion of this hospital's samples with a large presence of the *bla*_*NDM-1*_ gene. A matter of great concern is the association of multidrug-resistant phenotype with biofilm formation. This situation warranted stringent surveillance and adherence to infection prevention and control practices.

## 1. Introduction

Acinetobacter, a widely distributed, saprophytic bacterium in nature, has established itself as one of the most common nosocomial pathogen [1, 2]. Although different species of Acinetobacter are the potential to cause infection, 80% of infections are caused by Acinetobacter *baumannii*. Ease of survival even in adverse environments, ability to form biofilms on surfaces, and possession of many genes for antimicrobial resistance have made this bacterium an important pathogen. The potential ability of the bacterium to form biofilms in certain instances, indeed, provides a potential explanation for outstanding antibiotic resistance and survival properties in the harsh environment of hospitals, particularly in the intensive care setting [3–5].

Over the past few decades, its clinical importance had increased due to its ability to receive antimicrobial resistance factors [6, 7] through the transfer of plasmid or transposons that contained antimicrobial resistant genes, particularly in a hospital setting where usage of antibiotics are huge leading to selective pressure [8, 9]. Multidrug resistant (MDR) Acinetobacter species are defined as isolates resistant to the major three classes of antimicrobial agents - all penicillins and cephalosporins (including inhibitor combinations), fluoroquinolones, and aminoglycosides [7–11]. These strains are implicated in a variety of life-threatening infections such as ventilator-associated pneumonia (VAP), urinary tract infections, bloodstream infections, surgical site infections, and infections associated with medical devices, occurring especially in patients of intensive care units. Moreover, a significant correlation between biofilm formation and multidrug resistance has been attributed to the threat imposed by Acinetobacter to the current antibiotic era [8, 9, 12].

Diagnosis of multidrug-resistant Acinetobacter infection is a great challenge owing to the distribution of various species in relation to the type of infection, their antimicrobial profile, and biofilm-forming phenotype. Hence, from effective management and infection control perspectives, it is crucial to minimize the risk associated with Acinetobacter infection in a healthcare setting. This study was conducted to characterize the clinical Acinetobacter isolates with special reference to the detection of antimicrobial resistance, biofilm formation, and the presence of the *bla*_*NDM-1*_ gene.

## 2. Materials and Methods

A total of 324 Acinetobacter *species* isolates were obtained from various clinical specimens, and submitted to the Department of Microbiology, B. P. Koirala Institute of Health Sciences (BPKIHS) Dharan, Nepal. This study was conducted from September 2017 to August 2018. Ethical approval was obtained from the Institutional Review Committee of BPKIHS, Dharan, Nepal.

### 2.1. Identification of Acinetobacter Species

Direct microscopic examination of Gram-stained smear of all samples except blood were performed. Inoculation of samples onto appropriate culture media, incubation, and detection of growth after the recommended duration was carried out by standard microbiological techniques [13]. On blood agar suspected smooth, opaque colonies corresponding to nonlactose fermenting colonies on MacConkey and on CLED agar plates were presumed as Acinetobacter and processed further. Species identification of the genus Acinetobacter was carried out by several biochemical tests which included triple sugar iron (TSI) fermentation test, oxidase, indole, motility, urease, and arginine hydrolysis [14, 15].

### 2.2. Antimicrobial Susceptibility Test

An antibiotic sensitivity test was conducted on Mueller Hinton agar (MHA) by the Kirby Bauer disc diffusion method recommended by the Clinical and Laboratory Standard Institute (CLSI) guidelines [13]. Escherichia *coli* ATCC 25922 and Pseudomonas *aeruginosa* ATCC 27853 were used as control and tested along with the test strain. Antimicrobial drugs tested were piperacillin (100 *μ*g), ceftazidime (30 *μ*g), ceftriaxone (30 *μ*g), cefotaxime (30 *μ*g), cefepime (30 *μ*g), ciprofloxacin (5 *μ*g), imipenem (10 *μ*g), amikacin (30 *μ*g), and ampicilllin/sulbactum (10/10 *μ*g). Resistances to at least one antimicrobial agent in ≥3 antimicrobial classes were considered as multidrug resistance (MDR) [13].

### 2.3. Detection of ESBL Phenotype

According to the CLSI guidelines, probable ESBL-producing isolate had a zone of inhibition for ceftazidime (30 *μ*g) ≤ 22 mm and cefotaxime (30 *μ*g) ≤ 27 mm [13]. In order to confirm ESBL production, ceftazidime (30 *μ*g) and ceftazidime + clavulanate (30/10 *μ*g) discs were placed in Acinetobacter culture. Zones of inhibition were compared with the ceftazidime and cefotaxime discs alone and compared with the combined ceftazidime + clavulanate disc. An enhanced zone of the diameter of ≥5 mm in combination with clavulanate was confirmed isolates as ESBL [13].

### 2.4. Detection of Metallo-*β*-Lactamase Enzyme (MBL) Phenotype

#### 2.4.1. Combined Disc Diffusion Test

A combined disc diffusion test was employed to determine the MBL-producing phenotype in Acinetobacter. On the MHA plate lawn culture of Acinetobacter, imipenem disc (10 *μ*g) and imipenem disc with 10 *μ*l of 0.5 M EDTA were applied 20 mm apart from center to center. The zone of inhibition of >7 mm around the imipenem-EDTA disc compared to the imipenem disc alone classified the isolate as an MBL producer [16].

#### 2.4.2. Carbapenemase Production Test

Phenotypic detection of carbapenemase-producing MDR Acinetobacter was determined by a modified Hodge test [13]. First of all, an overnight broth culture of Escherichia *coli* ATCC 25922 was adjusted to 0.5 McFarland standards and spread on the dried surface of Mueller Hinton agar (MHA) plate by sterile cotton swab. After transitory drying, a 10 *μ*g imipenem (IMP) disc was placed at the center of the plate, and tested strains were streaked from the center to the periphery of the plate in four different directions. Following overnight incubation at 37°C, carbapenemase-positive isolates showed the distorted zone of inhibition, and a “clove leaf pattern” was observed due to carbapenemase production by isolates [13].

### 2.5. Molecular Detection of *bla*_*NDM-1*_ Gene

New Delhi metallo beta lactamase-1 (*bla*_NDM-*1*_) is a novel MBL that confers resistance to all *β*-lactam antibiotics with the exception of aztreonam. In this study, the multidrug resistant organisms were determined to have the *bla*_NDM -1_ gene by conventional PCR [17].

### 2.6. DNA Extraction of Bacterial Isolates

The overnight broth cultures were centrifuged at 3,500 rpm for 10 minutes at 4°C. Then, the supernatant was discarded and the pellet was washed twice with 5 ml phosphate buffered saline (PBS) followed by centrifugation. The pellet was resuspended in 1 ml PBS and centrifuged at 10,000 RPM for 10 minutes at 4°C. Finally, the supernatant was discarded and the remaining pellet was stored at −20°C till assayed [18].

The pellet was dissolved in 200 *μ*L of TE buffer. Then, the mixture was heated at 100°C for 10 minutes with shaky and rapidly transferred to an ice bath for 10 minutes. Centrifugation was performed at 13,000 rpm for 30 seconds at 4°C. Finally, 100 *μ*L of supernatant DNA was transferred to a new tube. The concentration and purity of the DNA were measured by Nanodrop 2000 spectrometer (Thermofisher) [17].

### 2.7. Polymerase Chain Reaction (PCR) for Detection of *bla*_NDM-1_

The *bla*_NDM-1_ gene-specific PCR was performed to detect *bla*_NDM-1_. PCR master mix was prepared in 25 *μ*L final volume which constituted 1X Qiagen PCR buffer, 2 mM MgCl_2_, 0.1 mg/ml BSA, 0.2 mM of dNTP mix, 0.8 *μ*M of each primer NDM1-F (5′-CAGCACACTTCCTATCTC-3′) and NDM1-R (5′-CCGCAACCATCCCCTCTT-3′), 0.5 Unit of Taq polymerase, and 2 *μ*L of DNA template. PCR amplification was carried out in Eppendorf Mastercycle ProS (Eppendorf, Germany) with (i) initial denaturation at 95°C for 5 minutes, (ii) 35 c ycles of denaturation at 94°C for 30 seconds, 55°C for 30 seconds, then at 72°C for 30 seconds, and (iii) a final extension at 72°C for 10 minutes. PCR water was used as a negative control, and the DNA from a bacterial culture with *bla*_NDM-1_ PCR positive result was considered as a positive control. After electrophoresis of amplified DNA in 2% agarose gel at 5 V/cm and ethidium bromide staining, the DNA band was visualized with UV exposure [19]. The sample was identified as *bla*_NDM-1_ PCR positive result if DNA band of 300 bp, as seen in the gel.

### 2.8. Detection of Biofilm Formation

Detection of biofilm formation was performed by the standard laboratory methods described elsewhere [20–23]. The bacterial isolates were grown overnight at 37°C in 5 ml of tryptic soy broth (TSB). Methicillin-sensitive*Staphylococcus aureus* (MSSA) ATCC-25923 and *P. aeruginosa* ATCC-27853 were used as negative and positive controls, respectively. Each well of a 96-wellflat-bottomed plates were filled with 200 *μ*L with overnight culture broth (0.5 McFarland standard diluted with 1% glucose + TSB). The plates were covered with lids and incubated aerobically for 24 hours at 37°C. After incubation, the content of each well was removed and washed three times with 300 *μ*L sterile phosphate-buffered saline (PBS; pH 7.2) in order to remove freely floating bacteria.

Succeeding every washing, the plates were drained in an inverted position. Adherent bacteria was fixed with 150 *μ*L of methanol for 20 min, those 96-well plate was emptied by simple flicking, and was left to air dry overnight at room temperature. The adherent biofilm layer formed in each 96-well plate's well were stained with 150 *μ*L of 2% Hucker crystal violet for 15 minutes. Excess stains were rinsed off by placing those 96-well plate under the running tap water until the release of stain got stopped. The 96-well plate were air-dried at room temperature. Then, 150 *μ*L of 95% ethanol was gently added in each well of the microtiter-plate in order to permit cell re-suspension. Again, the plate was incubated at room temperature for 30 minutes without shaking. The absorbance (*A*) of each solution, well stained with crystal violet, was measured at 570 nm using a microtiter-plate reader [23].

Interpretation of “*A*” measurements: the average “*A*” values were calculated for all tested strains and negative controls. The cut-off value (Ac) was calculated as three standard deviations (SD) above the mean “*A*” of the negative control. Ac = average “*A*” of negative control + (3 × SD of negative control). Strains were divided into four categories based upon “*A*” values: (a) *A* ≤ Ac = no biofilms producer, (b) Ac < *A* ≤ 2 × Ac = weak biofilm production (+), (c) 2 × Ac < *A* ≤ 4 × Ac = moderate biofilm production (++), and (d) 4 × Ac < *A* = strong biofilm production (+++) [23].

### 2.9. Statistical Analysis

Data were entered in MS Excel 2013 worksheet and statistical analysis were carried out by using *R* package version 0.55 [24]. The principle component analysis among the several factors such as MDR, MBL, _*bla*NDM_, and biofilms were carried out by using the “prcomp” function of the *R* stat package, correlation, and visualization of the plot were demonstrated by the ggbiplot package [25].

## 3. Results

Among 324 isolates of Acinetobacter, 167 (51.5%) were Acinetobacter *calcoaceticus-baumannii complex (Acb complex)* followed by 83 (25.6%) *A. lwoffii*, 38 (11.7%) *A. haemolyticus*, 30 (9.3%) *A. radioresistens,* and 6 (1.9%) *A. junii.*

Amongst those different specimens analyzed, *Acb complex* was the predominant species (Table 1).

In this study, 26% of the samples were obtained from the medical ward, 20% from ICU, 12% from OPD,11% from surgery and pediatrics, 6% from gynecology, 4% of emergency, NICU, and orthopedic department. *Acb complex* was predominant in ICU (76.7%).

### 3.1. Antimicrobial Susceptibility Testing

The resistance percentages of Acinetobacter in the descending order of frequency were cefepime 74.4% cefotaxime 73.8%, ceftriaxone 65.7%, ceftazidime 72.5%, ceftazidime + clavulanic acid 72.2%, piperacillin 65.7% ampicillin + sulbactam 36.7%, amikacin 44.7%, ciprofloxacin 50%, and imipenem 35.2%. *Acb complex* was found to have the highest drug-resistant phenotypes to analyze antibiotics with 57.4% being resistant to imipenem. For the Acb complex, cefotaxime was the antibiotic with the highest resistance frequency (94%), as for A. hemolyticus, it was 31 isolates out of 38 (82%). More than 50% of A. lwoffii and A. junii isolates were sensible to the investigated antimicrobials (Table 2). Acinetobacter isolates from ICU were more resistant to the antibiotics than those from other wards.

Among 324 isolates, 128 (39.5%) were MDR. Most of MDR were from patients in ICU 60.3% followed by OPD 43.5%, Ward 32.1%, and Emergency 20.0%. *Acb complex* had the highest rate of MDR phenotype as shown in Table 3.

**Figure 1:**
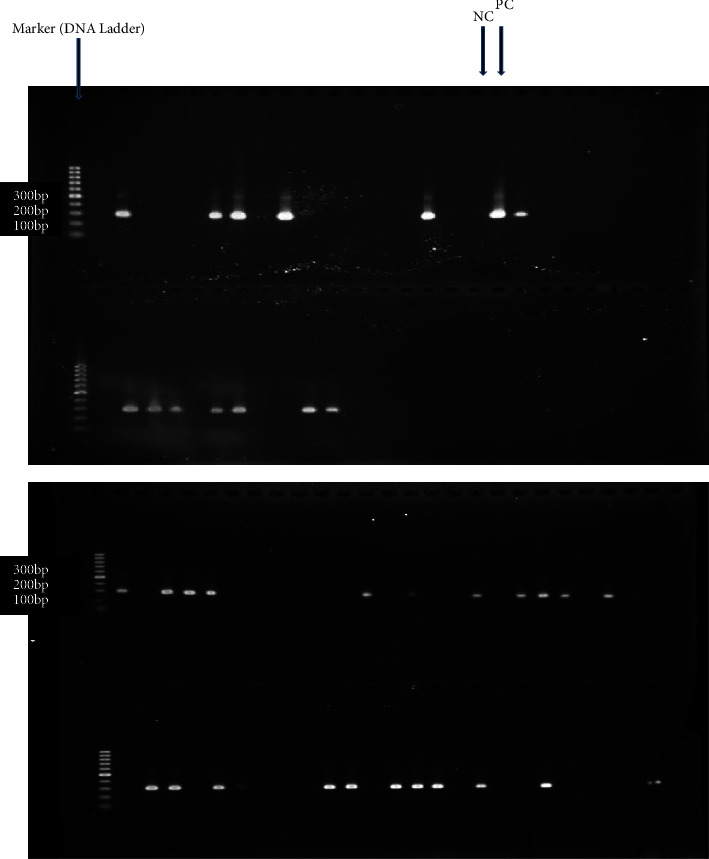
Agarose gel electrophoresis represents 300 bp *bla*_NDM-1_ gene in Acinetobacter *Species* (Amplicon size-300 bp).

*bla *_*NDM-1*_ gene was detected in 33 isolates. *bla*_NDM-1_ was carried in all species of Acinetobacter except in A. radioresistens. Out of those 33 isolates, *bla*_NDM-1_ isolates carried from various specimens included pus (64.3%), blood (62.5%), CSF (100%), ET-tubes (42.9%), ascetic fluid (33.3%), and urine (20%). All 33 *bla*_NDM-1_ producers of this study were XDR strains. PCR-amplification product of the *bla*_NDM-1_ gene was revealed in Figure 1.

### 3.2. Biofilm Formation

**Figure 2:**
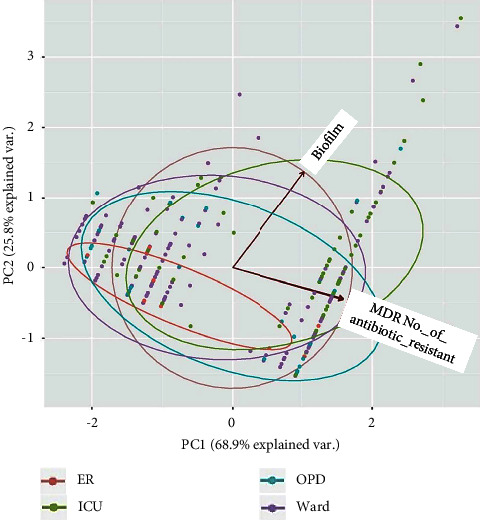
Principal component analysis (PCA) among isolates' origin, Biofilm formation, MDR, and a number of antibiotic resistance.

**Figure 3:**
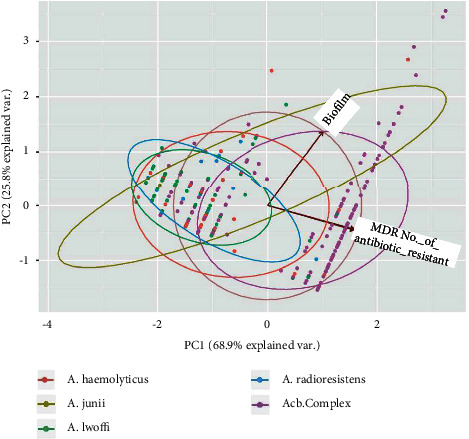
Principle component analysis (PCA) among Acinetobacter species, Biofilm formation, MDR, and a number of antibiotic resistance.

Among 324 isolates, biofilm production was detected in 121 (37.3%) isolates. Forty-five (37.2%) biofilm-forming isolates were obtained from the device, 36 (29.8%) from pus, 20 (16.5%) from blood, 18 (14.9%) from urine, and 7 (5.8%) from sputum. Biofilm production was found in all species as depicted in Table 3. About 60.3% of MDR and 64.4% biofilm-forming isolates were from ICU. Moreover, MDR phenotype and biofilm formation phenotypes were significantly associated (*p* value < 0.0001) whereas no association was determined among other virulence phenotypes such as ESBL, MBL, carbapenemase, and *bla*_NDM-1_. The principal component analysis (PCA) among Acinetobacter isolates, origin, biofilm formation, MDR, and number of antibiotic resistance in the diagram showed the findings that the circle is more closer to Y – axis which represents biofilm whereas the circles represents the origin of isolates and Acinetobacter species in Figure 2 and Figure 3, respectively. Similarly, biofilm formations were consistently found in isolates from ICU (Figure 2) and *Acb complex* (Figure 3) as shown by principal component analysis.

## 4. Discussion

Acinetobacter is one of the notorious nosocomial pathogen and its tendency to develop resistance against antimicrobial drugs is an important rationale for infection control at Health care facility.

Among five Acinetobacter species, Acb (Acinetobacter calcoaceticus-A. baumannii) complex was one of the most predominating species (51.5%) in this study, which was comparable to the findings of other studies [15]. It suggests Acb complex has more survival rate even in an unfavorable environment and causes hospital acquired infection. About 20% of isolates were obtained from ICU which is similar to findings reported in the previous study from Nepal [26]. This indicates that ICU could be the most important location for the colonization and survival of Acinetobacter in at hospital environment [5, 27]. ICU patients usually require a prolonged hospital stay, need repeated invasive procedures and utilizes various devices for life support, and frequently receives treatment with broad-spectrum antimicrobials. Most of the sample isolates were of the cases of sepsis from the ICU. Previous antimicrobial therapy, medical devices, and prolonged hospitalization are the known risk factors for bacteremia in such patients [28].

Resistance to cefepime (74.4%) and cefotaxime (73.8%) were detected in 74.4% and 73.8% of isolates respectively, followed by ceftazidime (72.5%), ceftriaxone (65%), and piperacillin 65%. It was found that the isolates resistance to amikacin was 44.7% and to ciprofloxacin 50.0% which were consistent with other reports [28, 29]. This indicates that Acinetobacter species have intrinsic and/or easily acquired mechanisms of resistance against many of the available antimicrobial agents making this pathogen one of the most significant microbial challenges for the current period.

Although carbapenem was the first-line drug against Acinetobacter infection in the late 1990s, carbapenem-resistant strains are increasingly reported worldwide [10]. Among the ICU isolates, 42.5% were sensitive to ampicillin/sulbactam and imipenem. The finding on imipenem resistance of 35.2% (114/324) poses a concern.

In this study, 128 (39.5%) isolates were determined as multidrug resistant (MDR), in which it was found that all species were MDR strains. Acinetobacter appeared to have the propensity to develop antibiotic resistance rapidly, as a consequence of prolonged antibiotic exposure. Hence, the increasing trend of Acinetobacter MDR strains were reported globally [30, 31].

In this study, 235 (72.5%) of the strains were ceftazidime resistant, and 13 (4.0%) of them demonstrated ESBL production by double disc synergy test which disagree with other reports [30, 32]. Since the antimicrobial susceptibility pattern could be variable depending on several factors, the surveillance studies have a crucial role in deciding the therapy against Acinetobacter infection [15]. In this study Among MDR isolates, 10.7% had demonstrated carbapenemase production by the MHT method. The MHT and combined disc diffusion tests with Imipenem and EDTA have been extensively used as a phenotypic assay for the detection of carbapenemase and MBL enzyme as it is a simple test to perform. MHT had been found to be sensitive in the detection Ambler class A (KPC) and class *D* (OXA-48) whereas its sensitivity is very low for NDM-1 producer which was demonstrated in this study as well [15, 33]. Whereas, there is high sensitivity but low specificity rate of combined disc test for detection of MBL production by a phenotypic method which result may increase the false positive rate of MBL [34].

The gold-standard for the identification of carbapenemase production and detection of the *bla*_NDM-1_ gene is PCR assay [35]. Moreover, other assay such as loop-mediated isothermal amplification [36] had been developed for the detection of the *bla*_NDM-1_ gene. In this study, NDM-producing Acinetobacter isolates was 10.2% (33/324) which was consistent with the study, carried out in western Nepal [37]. The evidence of Acinetobacter with the presence of the *bla*_NDM-1_ gene had already been reported worldwide [38, 39]. In this study, 91% of NDM producers were resistant to second and third-generation cephalosporins. Moreover, 25.8% (33/128) of *bla*_NDM-1_ gene-containing isolates had MDR phenotype. However, Imipenem was the most effective antibiotic in the study with only 35.2% imipenem resistance.

The biofilm-forming phenotype of Acinetobacter was determined by 96 well plate assay and were found 37.3% of isolates were biofilm formers of which was inconsistent with the previously reported 73.7% [40]. Moreover, 65.3% of biofilm-forming isolates had significantly associated with multidrug resistant phenotype. Isolates with the biofilm producer were *Acb complex* (56.2%) followed by A. lwoffii (14.5%), A. haemolyticus (21.1%), A. radioresistens (20%) and A. Junii (16.7%). Biofilm-forming isolates were found mostly on samples from medical devices (37.2%) followed by pus (29.8%) which was reliable with most of the studies, that reported more than 90% biofilm-producing isolates from medical devices as well as showed multidrug resistance pattern [41, 42]. In addition, the biofilm-forming isolates were predominantly in ICU (64.4%) followed by wards (31.1%) which was also consistent with other studies and it suggested that medical devices help to colonize prior to the development of biofilm formation [43]. Among the biofilm formers in this study, resistance to imipenem at 62% (75/121) was higher than the 46.7% reported by Yadav et al. [5]. This study reported that the clinical Acinetobacter isolates from ICU had both phenotype biofilm producers and multidrug resistance [3, 4, 42]. Moreover, the association between biofilm producers and antibiotics had been demonstrated by multiple studies. The rationale is that, once devices are colonized, the biofilms that develop share fundamental characteristics of all bacterial biofilms, and the cells within the biofilm were protected by the extracellular matrix, that protective material could decrease the effectiveness of both antibiotics and host defense mechanisms [44, 45]. However, it was difficult to identify the antibiotic resistant pattern of biofilm-producer microorganism by manual AST, because planktonic bacterial cells were used for susceptibility tests. In addition, antibiotics resistance might be even higher than observed in the present study, as bacteria without a molecular basis of resistance (susceptible in vitro) could be resistant in a biofilm environment.

Although there were innovative antibiofilm therapeutics which included combining APDT with antibiotics, plant extracts, or biofilm-disrupting enzymes that could assist in managing such cases. It helped to increase the sensitivity of the microorganism to antibiotic therapies by violating the structure of the biofilm or disturbing the communication between populations of microorganisms in the biofilm [46].

However, different results were seen in different studies due to variations as of geography, arrangement of specimens in the study groups, condition of the patient, and use of antibiotic.

The data from this study demonstrated that Acinetobacter species were resistant to many of the available antimicrobial agents, making those nosocomial pathogens as one of the most significant microbial challenges to have the control in future.

## 5. Conclusion

The clinical isolates of Acinetobacter in this setting were multidrug-resistant MBL producers with *blaNDM-1* gene and biofilm formers. In addition, there is evidence that the biofilm formation is a potential marker to determine the multidrug resistant (MDR) phenotype. These isolates have been proven to cause nosocomial infection in healthcare settings and are challenging to treat. Therefore, a consolidated effort by all healthcare providers by strict implementation of infection prevention and control activities, early diagnosis, and antibiotic stewardship are recommended to reduce the burden of antimicrobial resistance on patients and health facilities.

## Figures and Tables

**Table 1:** *Acinetobacter* species from different clinical samples (*n* = 324).

Specimen types	*Acb complex n* = 167 (51%)	A. lowffi *n* = 83 (26%)	*A. haemolyticus n* = 38 (12%)	*A. radioresistens n* = 30 (9%)	*A. junii n* = 6 (2%)	Total
Blood	33 (39%)	27 (32%)	14 (17%)	9 (11%)	1 (1%)	84 (26%)
Pus	61 (74%)	11 (13%)	3 (4%)	6 (7%)	1 (1%)	82 (25%)
Urine	15 (31%)	17 (35%)	6 (12%)	10 (20%)	1 (2%)	49 (15%)
Sputum	12 (60%)	3 (15%)	3 (15%)	2 (10%)	—	20 (6%)
Endotracheal aspirate	31 (85%)	2 (5%)	2 (5%)	2 (5%)	—	37 (11%)
Exudate body fluid	6 (29%)	12 (56%)	1 (5%)	—	2 (10%)	21 (6.2%)
Central venous catheter	3 (33%)	2 (22%)	4 (44%)	—	—	9 (3%)
CSF	3 (43%)	1 (14%)	3 (43%)	—	—	7 (2%)
HVS	—	2 (50%)	1 (25%)	1 (25%)	—	4 (2%)
Nasal swab	1 (14%)	4 (57%)	0	—	1 (14%)	7 (1%)
Tissue	1 (50%)	—	1 (50%)	—	—	2 (0.6%)
Semen	—	2 (67%)	1 (33%)	—	—	3 (1%)

Note. CSF: cerebrospinal fluid and HVS: high vaginal swab.

**Table 2:** Antibiotic resistance profile of isolated Acinetobacter species.

Antibiotics (*n* = no. of resistant isolates)	Acb complex (*a* = 167)	A. lwoffii (*a* = 83)	A. hemolyticus (*a* = 38)	A. junii (*a* = 6)	A. radioresistens (*a* = 30)
Piperacillin *n* = 213 (65.7%)	143 (85.6%)	29 (34.9%)	25 (65.7%)	3 (50%)	13 (43.3%)
Ampicillin + sulbactam *n* = 119 (36.7%)	97 (58%)	4 (4.8%)	11 (28.9%)	1 (16.6%)	6 (20%)
Ceftazidime + Clavulanic acid *n* = 234 (72.2%)	149 (89.2%)	35 (42.1%)	30 (78.9%)	2 (33.3%)	18 (60%)
Ceftazidime *n* = 235 (72.5%)	150 (89.8%)	36 (43.3%)	30 (78.9%)	2 (33.3%)	17 (56.6%)
Cefepime *n* = 241 (74.4%)	155 (92.8%)	35 (42.1%)	30 (78.9%)	3 (50%)	18 (60%)
Cefotaxime *n* = 239 (73.8%)	157 (94%)	32 (38.5%)	31 (81.5%)	2 (33.3%)	17 (56.6%)
Ceftriaxone *n* = 213 (65.7%)	147 (88%)	23 (27.7%)	29 (76.3%)	2 (33.3%)	12 (40%)
Imipenem *n* = 114 (35.2%)	96 (57.4%)	5 (6%)	9 (23.6%)	1 (16.6%)	3 (10%)
Amikacin *n* = 145 (44.7%)	117 (70%)	9 (10.8%)	12 (31.5%)	1 (16.6%)	6 (20%)
Ciprofloxacin *n* = 162 (50.0%)	127 (76%)	16 (19.2%)	11 (28.9%)	1 (16.6%)	7 (23.3%)

**Table 3:** Acinetobacter species in relation to various types of resistance mechanisms and biofilm formation.

Characteristic of isolates (*a* = no of isolates)	*Acb complex*(*n* = 167)	*A. lwoffii* (*n* = 83)	*A. haemolyticus*(*n* = 38)	*A. radioresistens*(*n* = 30)	*A. junii* (*n* = 6)
MDR (*a* = 128)	111 (66.5%)	4 (4.8%)	8 (21.1%)	4 (13.3%)	1 (16.6%)
ESBL (*a* = 13)	10 (5.9%)	—	1 (2.6%)	2 (6.6%)	—
Carbapenemase (*a* = 12)	10 (5.9%)	—	2 (5.2%)	—	—
MBL (*a* = 70)	56 (33.5%)	3 (3.6%)	7 (18.4%)	3 (10%)	1 (16.7%)
*bla *_NDM-1_ genotype (*a* = 33)	28 (16.8%)	2 (2.4%)	2 (5.2%)	—	1 (16.7%)
Biofilm (*a* = 121)	94 (56.2%)	12 (14.5%)	8 (21.1%)	6 (20%)	1 (16.7%)

Note. MDR:multidrug resistant, ESBL: extended spectrum beta-lactamase, MBL: metallo-*β*-lactamase, and *bla*_NDM-1_= New Delhi metallo-beta-lactamase 1.

## Data Availability

This is a hospital-based study. Samples were collected during the routine diagnosis and genotyping procedure. Therefore, the data for the analysis will available upon the request from the corresponding author or head of the department of Microbiology (hod.microbiology@bpkihs.edu), BP Koirala Institute of Health Sciences, Dharan, Nepal.
